# Effects of Exercise Training on Growth and Differentiation Factor 11 Expression in Aged Mice

**DOI:** 10.3389/fphys.2019.00970

**Published:** 2019-07-31

**Authors:** Minjung Lee, Satoshi Oikawa, Takashi Ushida, Katsuhiko Suzuki, Takayuki Akimoto

**Affiliations:** ^1^Faculty of Sport Sciences, Waseda University, Saitama, Japan; ^2^Division of Regenerative Medical Engineering, Center for Disease Biology and Integrative Medicine, Graduate School of Medicine, The University of Tokyo, Bunkyo, Japan

**Keywords:** exercise, skeletal muscle, aging, sarcopenia, growth and differentiation factor 11

## Abstract

Exercise training is considered an effective way to prevent age-related skeletal muscle loss. However, the molecular mechanism has not been clarified. Growth and differentiation factor 11 (GDF11) has been controversially considered a regulator of skeletal muscle aging. In this study, we examined whether GDF11 is associated with skeletal muscle aging and the effects of exercise training on age-related skeletal muscle loss. First, we observed that *Gdf11* mRNA and protein expression levels in young (5-month-old, *n* = 6) and aged (22-to 26-month-old, *n* = 5) mice were not significantly different. Aged mice were then divided into sedentary (*n* = 5) and exercise (*n* = 6) groups. The exercise group performed moderate-intensity treadmill running for 6 weeks. Treadmill exercise training increased *Gdf11* mRNA expression in the soleus muscle, but its protein expression was not altered. In contrast, the GDF11 level in the plantaris muscle was not changed at either the mRNA or protein level. Collectively, our data demonstrate that GDF11 levels do not change during aging, and that treadmill exercise training increased *Gdf11* mRNA expression in a predominantly slow-twitch muscle.

## Introduction

Aging increases an animal’s vulnerability to adverse health outcomes, including death. In the human body, progressive muscle loss and dysfunction, termed “sarcopenia,” is one of the many distinct age-related changes. It has been reported that reductions in muscle mass and function with aging can reduce the quality of life in the elderly and even increase mortality, regardless of other risk factors (Roubenoff and Hughes, 2000; Landi et al., 2013). Because the aging population is increasing dramatically in most developed countries, it is necessary to clarify the mechanism of sarcopenia.

Many studies have reported that increased physical activity improves sarcopenia (e.g., Cruz-Jentoft et al., 2014). Resistance exercise training not only increases skeletal muscle mass, but also improves the muscle strength and contractile function of muscle fibers, even in the elderly (Trappe et al., 2000; Holm et al., 2008; Verdijk et al., 2009). Long-term endurance exercise training, such as cycling (Harber et al., 2012) and running (Schwartz et al., 1991), also induces muscle hypertrophy in the elderly. Whether endurance exercise is an effective way to reduce muscle loss in sarcopenia is controversial (Short et al., 2004; Weiss et al., 2007), but people who regularly perform endurance exercise show lower rates of muscle atrophy (Sugawara et al., 2002), indicating that endurance exercise may repress sarcopenia. However, the molecular mechanisms underlying these effects of exercise on sarcopenia are still unclear.

The amino acid sequence of growth and differentiation factor 11 (GDF11), a member of the transforming growth factor *β* (TGF-β) family, is almost 90% identical to that of myostatin (GDF8) in its mature forms. Myostatin is exclusively expressed in skeletal muscle and has been shown to be a powerful inhibitor of muscle growth (McPherron et al., 1997). In contrast, GDF11 is expressed in a broad range of tissues and has been reported to function in the developmental processes of other organs, such as the axial skeleton (McPherron et al., 2009) and brain (Wu et al., 2003). It has been reported that, after binding to its receptors, myostatin regulates muscle mass through SMADS signaling pathway (Sartori et al., 2013). Because GDF11 and myostatin share several receptors (Lee and McPherron, 2001; Oh et al., 2002), it has been suggested that they are functionally redundant (McPherron et al., 2009). However, the recent proposition that GDF11 is a possible regulator of skeletal muscle aging is controversial (Sinha et al., 2014; Egerman et al., 2015). Sinha et al. reported that a reduction in GDF11 in serum with aging caused skeletal muscle dysfunction and that daily injections of recombinant GDF11 in aged mice reversed the aging phenotypes (Sinha et al., 2014). However, shortly after that paper was published, another study (Egerman et al., 2015) revealed that the antibody used in the aforementioned study also detected myostatin. They also showed that GDF11 levels rose with age in both mice and humans using an immunoassay specific to GDF11 (Egerman et al., 2015). Furthermore, daily injections of GDF11 reduced the muscle regeneration capacity (Egerman et al., 2015). However, again, the antibody used in that study was later found to be nonspecific for GDF11 (Poggioli et al., 2016). A recent study that used LC–MS/MS to distinguish between GDF11 and myostatin showed that GDF11 does not decline with age in humans, although myostatin was lower in older males than in younger ones (Schafer et al., 2016). Although the debate remains lively, GDF11 may regulate age-related muscle loss and be involved in the mechanism by which exercise attenuates sarcopenia. In this study, we investigated whether GDF11 levels in skeletal muscle changed in aged mice after treadmill running training for 6 weeks.

## Materials and Methods

### Animals and Exercise Protocol

Aged (22- to 25-month-old, *n* = 11) and young (5-month-old, *n* = 6) male C57BL6/J mice were purchased from Clea Japan, Inc. (Tokyo, Japan). All mice were housed in cages in a temperature-controlled (21°C) environment under a 12-h light/12-h dark cycle, with free access to food and water, according to the Guideline for Experimental Animal Care issued by the Prime Minister’s Office of Japan. The aged mice were housed individually in cages and randomly divided into two groups: an exercise training group (*n* = 6) and a sedentary group (*n* = 5). The exercise training group performed treadmill running 5 days a week for 6 weeks. Before exercise training, the mice were accustomed to the two-lane enclosed treadmill equipment (MELQUEST, Toyama, Japan) once by running for 10 min at a speed of 10 m/min with a 5% incline, during the acclimation period. The exercise protocol was modified from a previous study (Sakakima et al., 2004). The treadmill was initially set at a speed of 10 m/min at a 5% incline and the speed was gradually increased by 1.0–1.2 m/min a day to 16 m/min in week 6 (Supplementary Table S1). The incline was also increased from 5 to 10% on day 3 of the training period. Instead of electrical stimulation, the mice were gently touched with a paintbrush to encourage them to run. All the aged mice were sacrificed 2 days after the final exercise session to avoid the acute effects of exercise. The young mice were sacrificed at 5 months of age. All of the mice were sacrificed under anesthesia with inhalant isoflurane and their skeletal muscles (plantaris and soleus) were harvested. The muscles were immediately transferred into the appropriate buffer or embedded in O.C.T. compound. All samples were stored at −80°C until analysis. All the animal protocols were approved by the Animal Care and Use Committee of the University of Tokyo and Waseda University.

### Semiquantitative Reverse Transcription-Polymerase Chain Reaction

Total RNA was extracted from the skeletal muscle tissues with ISOGEN2 (Wako, Osaka, Japan), according to the manufacturer’s instructions. The concentration of total RNA was determined with a NanoDrop spectrophotometer (Nano-Drop ND-1000, Thermo Scientific, MA, USA) and 1 μg of RNA was reverse transcribed with the ReverTra Ace qPCR RT Kit (Toyobo, Osaka, Japan). The cDNA products were stored at −20°C until analysis. Ex Taq HS (TaKaRa, Osaka, Japan) was used for the PCRs with the following gene-specific primers: mouse *Gdf11*, 5′-AGCATCAAGTCGCAGATCCT-3′ and 5′-GGCCTTCAGTACCTTGGTGA-3′; *Gapdh*, 5′-GACCCCTTCATTGACCTCAAC-3′ and 5′-TAAGCAGTTGGTGGTGCAGGA-3′. *Gapdh* was used as the internal standard. The PCRs were performed in a Veriti 96 well Thermal Cycler (Applied Biosystems, MA, USA). The thermal protocol for PCR was: (1) 2 min of denaturation at 95°C; (2) 15 s of denaturation at 95°C, 15 s of annealing at 60°C, and 30 s of extension at 72°C (this step was repeated 35 times for *Gdf11* and 25 times for *Gapdh*); (3) 7 min of extension at 72°C. The reaction was held at 10°C until electrophoresis. The PCR products were separated electrophoretically for 50 min on 1–2% of agarose gels (1–2% agarose diluted in Tris-acetate-EDTA buffer with 0.01% ethidium bromide). The gels were transferred to a LAS-3000 imaging system (Fuji Film, Japan) and fluorescent images were acquired under UV light. The ImageJ software (National Institutes of Health, Bethesda, MD, USA) was used for image quantification.

### Western Blotting Analysis

The dissected muscle tissues were immediately transferred to complete protein-loading buffer and homogenized with a glass homogenizer. The complete protein-loading buffer contained 50 mM Tris-HCl (pH 6.8), 1% SDS, 10% glycerol, 20 mM dithiothreitol, 127 mM 2-mercaptoethanol, 0.01% bromophenol blue, protease inhibitors (Roche, Switzerland), and phosphatase inhibitors (Sigma-Aldrich, MO, USA). The soleus and plantaris muscles were homogenized with 200 and 400 μl of the buffer, respectively. The muscle homogenates were transferred to 1.5-ml microfuge tubes, denatured for 5 min at 95°C, and centrifuged for 10 min at 12,000 × *g*. The protein concentrations of the homogenates were measured with the RC DC Protein Assay Kit (Bio-Rad, CA, USA). The total proteins (60 μg) were loaded onto 12% gels and separated with sodium dodecyl sulfate-polyacrylamide electrophoresis (SDS-PAGE) for 1.5 h at 100 V. After SDS-PAGE, the proteins were transferred to a nitrocellulose membrane (Hybond ECL, GE Healthcare, UK) at 100 V for 1 h in a chamber filled with transfer buffer. Ponceau S solution was used to check the transferred proteins on the membrane. Nonspecific protein binding was blocked by incubating the membranes with 5% skim milk in Tris-buffered saline containing 0.05% Tween 20 (TBST) for 1 h at room temperature. The membranes were incubated with following primary antibodies: two anti-GDF11 antibodies purchased from Abcam (ab124721, rabbit monoclonal antibody, diluted 1:100, for 1 h at 37°C) or R&D Systems (MAB19581, mouse monoclonal antibody, diluted 1:500, overnight at 4°C), and two anti-GAPDH antibodies purchased from Millipore (MAB374, mouse monoclonal antibody, diluted 1:1000, overnight at 4°C) and Cell Signaling Technology (#2118, rabbit monoclonal antibody, diluted 1:1000, overnight at 4°C). After the membranes were washed three times with TBST, they were incubated with the following secondary antibodies: ECL™ anti-rabbit IgG HRP-linked F(ab’)2 fragment (GE Healthcare; diluted 1:2000) or mouse IgG κ light chain binding protein conjugated with horseradish peroxidase (HRP) (m-IgGκ BP-HRP, Santa Cruz Biotechnology, sc-516,102; diluted 1:2000). The signals were immunodetected with Amersham ECL Prime Western Blotting Detection Reagent (GE Healthcare) using the LAS-3000 imaging system. The signals were quantified with the ImageJ software.

### Histological Analysis

The plantaris and soleus muscles were embedded in a plastic mold covered with O.C.T. compound and transferred to precooled isopentane at −80°C. The O.C.T.-embedded muscles were cut transversely into 10-μm sections with a cryostat (Leica, Germany) at −20°C and immediately collected on glass slides. The sections were fixed on ice with 4% paraformaldehyde/phosphate-buffered saline (PBS) for 3 min and permeabilized with 0.3% Triton X-100/PBS for 10 min. Monoclonal antibodies directed against myosin heavy chain I (MyHC I; BA.F8), MyHC IIa (SC.71), and MyHC IIb (BF.F3) were diluted 1:25, 1:250, and 1:25, respectively in 5% normal goat serum (NGS)/PBS and used as the primary antibodies for muscle-fiber typing. The three primary antibodies were obtained from the German Collection of Microorganisms and Cell Cultures (DSMZ, Germany). A mouse anti-dystrophin antibody (D8043; Sigma-Aldrich) was diluted 1:100 in 5% NGS/PBS and used to identify the muscle-fiber shapes. Anti-mouse IgG2b–Alexa 405 (for MyHC I), anti-mouse IgG1–Alexa 488 (for MyHC IIa and dystrophin), and anti-mouse IgM–Alexa 549 (for MyHC IIb) were all diluted 1:100 in 5% NGS/PBS and used as the secondary antibodies. All the secondary antibodies and NGS were purchased from Jackson ImmunoResearch Laboratories (PA, USA). Images of the immunostained cross-sections were taken under an IX-70 fluorescence microscope (Olympus, Japan) with a digital camera (DS-Ri1, Nikon, Japan). The cross-sectional areas (CSAs) were measured using ImageJ software.

### Statistics

All data are presented as means ± SD. The effects of age (young sedentary mice vs. aged sedentary mice) and exercise training (aged exercised mice vs. aged sedentary mice) were tested with one-way ANOVA, followed by Dunnett’s (two-tailed) *post hoc* test (aged sedentary group). All statistical analyses were performed with IBM SPSS Statistics 22 (IBM, USA).

## Results

### Aging Induces Loss of Muscle Mass

**Figure 1:**
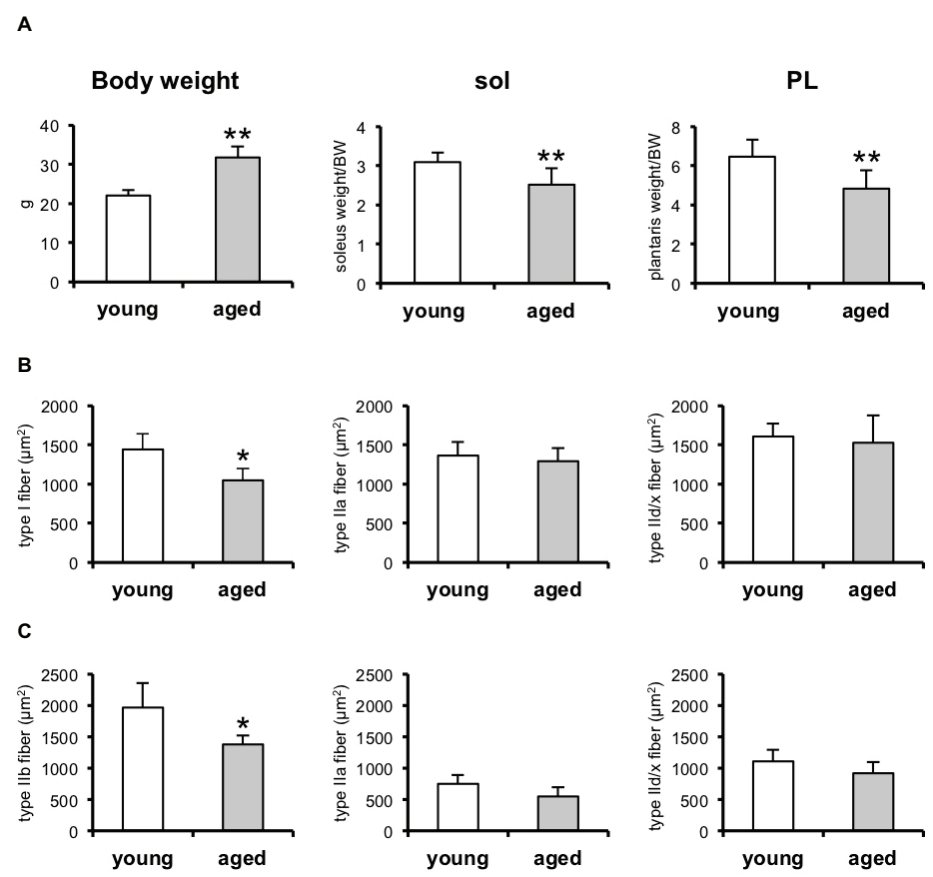
Skeletal muscle weights and cross-sectional areas (CSAs) of skeletal muscles in young and aged mice. **(A)** Bodyweight, soleus (sol) and plantaris (PL) muscle weights relative to bodyweight (BW). Young, *n* = 6; aged, *n* = 6. **(B)** CSA of type I, IIa, and IId/x in soleus muscles. Young, *n* = 6; aged, *n* = 5. **(C)** CSA of type IIb, IIa, and IId/x in plantaris muscles. Young, *n* = 6; aged, *n* = 5. Data are presented as means ± SD. ^**^p < 0.01, ^*^p < 0.05 vs. the young group.

**Figure 2:**
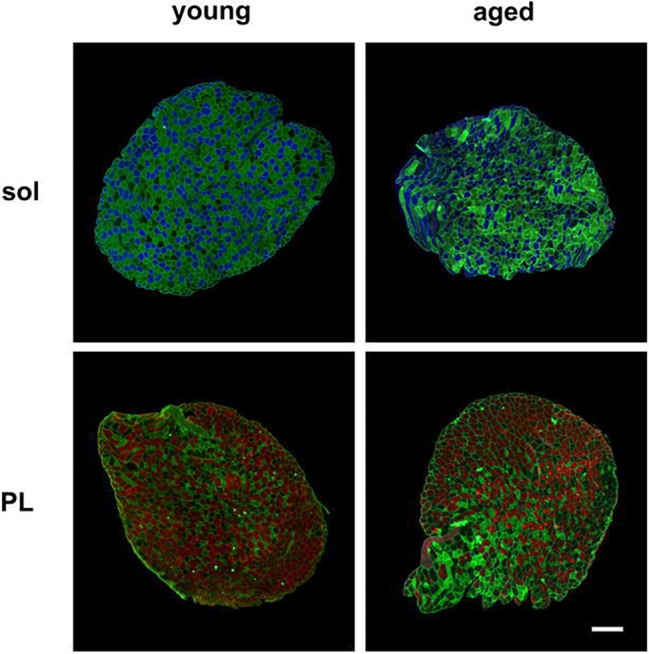
Representative images of muscle-fiber typing of soleus and plantaris muscles. Muscle sections were stained with antibodies directed against myosin heavy chains. Indirect immunofluorescent staining of type I (blue), type IIb (red), type IIa (green), and type IId/x (no staining) fibers is shown in the soleus (sol) and plantaris (PL) muscles of the young and aged mice. Scale bar: 200 μm.

We first observed that a loss of muscle mass had occurred in the aged mice (Figure 1A). We examined two skeletal muscles, the plantaris and soleus, that are composed by different fibers (plantaris, fast-twitch muscle fibers; soleus, slow-twitch muscle fibers; Figure 1A). We also observed the CSAs of each muscle-fiber type composing soleus (MyHC I, IIa, and IId/x; Figure 1B) and plantaris (MyHC IIa, IId/x, and IIb; Figure 1C) with indirect immunofluorescent staining (Figure 2). MyHC I in the soleus and MyHC IIb in the plantaris were significantly reduced in the aged mice (Figures 1B,C).

### Growth and Differentiation Factor 11 Expression in Skeletal Muscle Does Not Change With Aging

**Figure 3:**
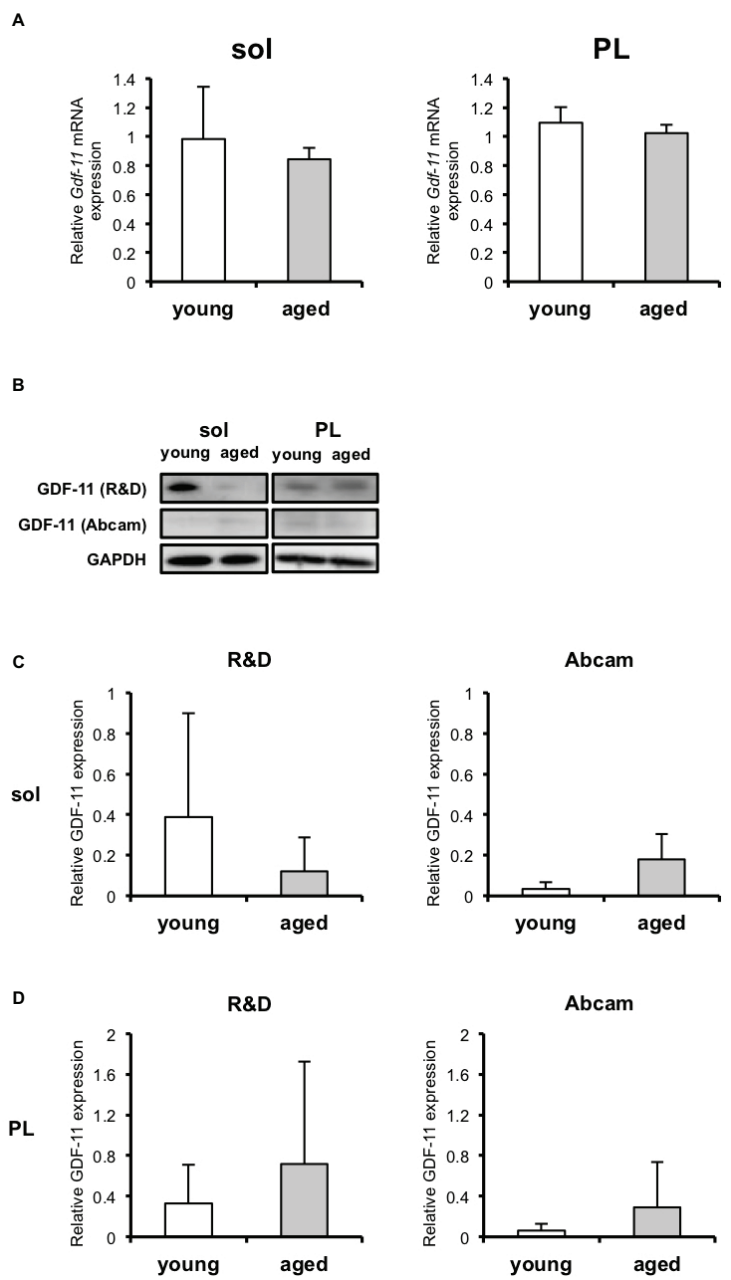
GDF11 expression in skeletal muscles of young and aged mice. **(A)** *Gdf11* mRNA levels in soleus (sol) and plantaris (PL) of young (*n* = 6) and aged (*n* = 5) mice. **(B)** GDF11 protein detected by western blotting using two different antibodies purchased from R&D Systems and Abcam. **(C)** Quantitative data for GDF11 protein levels in soleus and plantaris of young (*n* = 6) and aged (*n* = 4) mice, detected with the R&D Systems anti-GDF11 antibody. **(D)** Quantitative data for GDF11 protein levels in soleus and plantaris muscles of young (*n* = 6) and aged (*n* = 5) mice detected with the Abcam anti-GDF11 antibody. The changes in GDF11 expression with aging at both the mRNA and protein levels were not statistically significant. Data are presented as means ± SD.

We next determined whether aging affected the GDF11 levels in the skeletal muscles. We observed that none of the differences in *Gdf11* expression between skeletal muscles from young and aged mice were statistically significant at the mRNA level (Figure 3A). Because the detection of GDF11 protein has been controversial (Egerman et al., 2015; Smith et al., 2015), we performed western blotting with two different antibodies purchased from R&D Systems and Abcam (Figure 3B) that were used in previous studies (Sinha et al., 2014; De Domenico et al., 2017). The differences in the GDF11 protein levels in the mice skeletal muscles of the young and aged, detected with both the R&D (Figure 3C) and Abcam (Figure 3D) antibodies, were not statistically significant.

### Six Weeks of Low-Intensity Treadmill Running Training Had Little Effect on Skeletal Muscle

**Figure 4:**
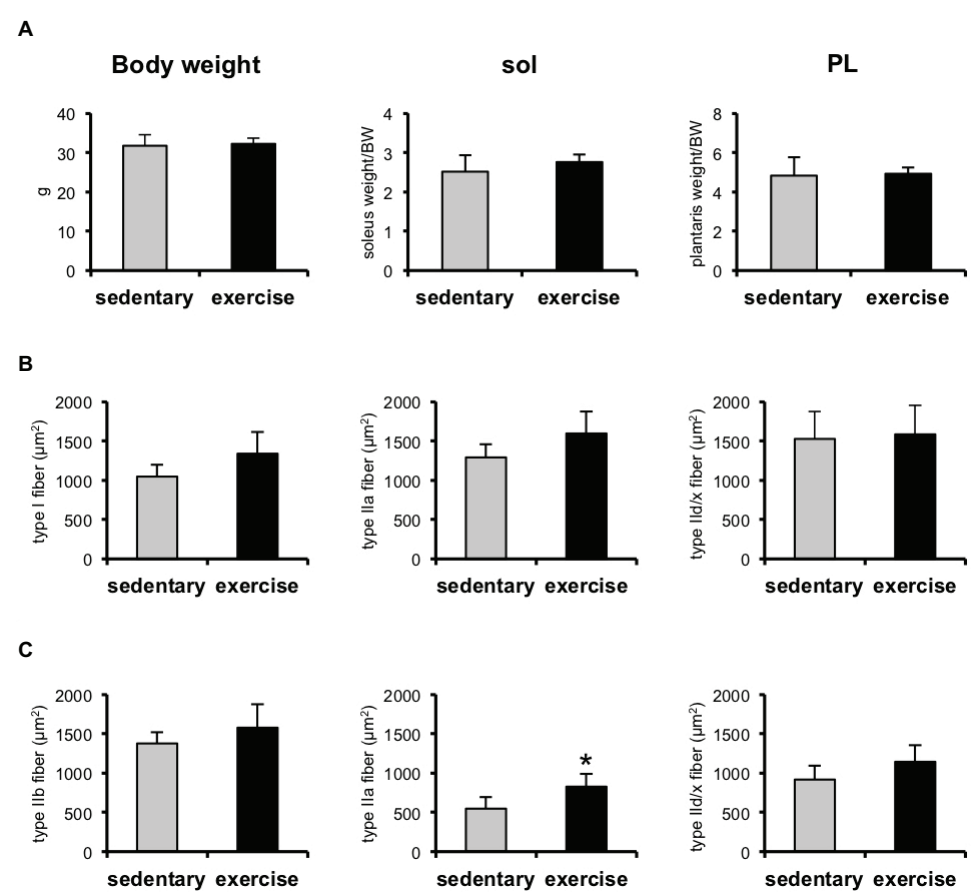
Skeletal muscle weights and cross-sectional areas (CSAs) of skeletal muscles in aged sedentary and aged exercised mice. **(A)** Bodyweight and soleus (sol) and plantaris (PL) muscle weights of aged sedentary (*n* = 4) and aged exercised mice (*n* = 6). **(B)** CSAs of type I, IIa, and IId/x in soleus muscles of aged sedentary (*n* = 4) and aged exercised mice (*n* = 6). **(C)** CSAs of type IIb, IIa, and IId/x in plantaris muscles of aged sedentary (*n* = 4) and aged exercised mice (*n* = 6). Data are presented as means ± SD. ^*^p < 0.05 vs. the sedentary group.

To investigate whether exercise training prevents aging-induced muscle loss, some mice were trained on a treadmill for 6 weeks with moderate-intensity exercise. We observed that the CSAs of MyHC IIa in the plantaris (Figure 4C) were increased by exercise training, whereas the CSAs of the other MyHC fibers composing the soleus (Figure 4B) and PL (Figure 4C) did not change. Despite the increase in MyHC IIa in the plantaris, the exercise training conducted in this study did not induce an increase in muscle mass (Figure 4A).

### *Gdf11* mRNA Increased in Slow Muscle in Response to Treadmill Running for 6 Weeks

**Figure 5:**
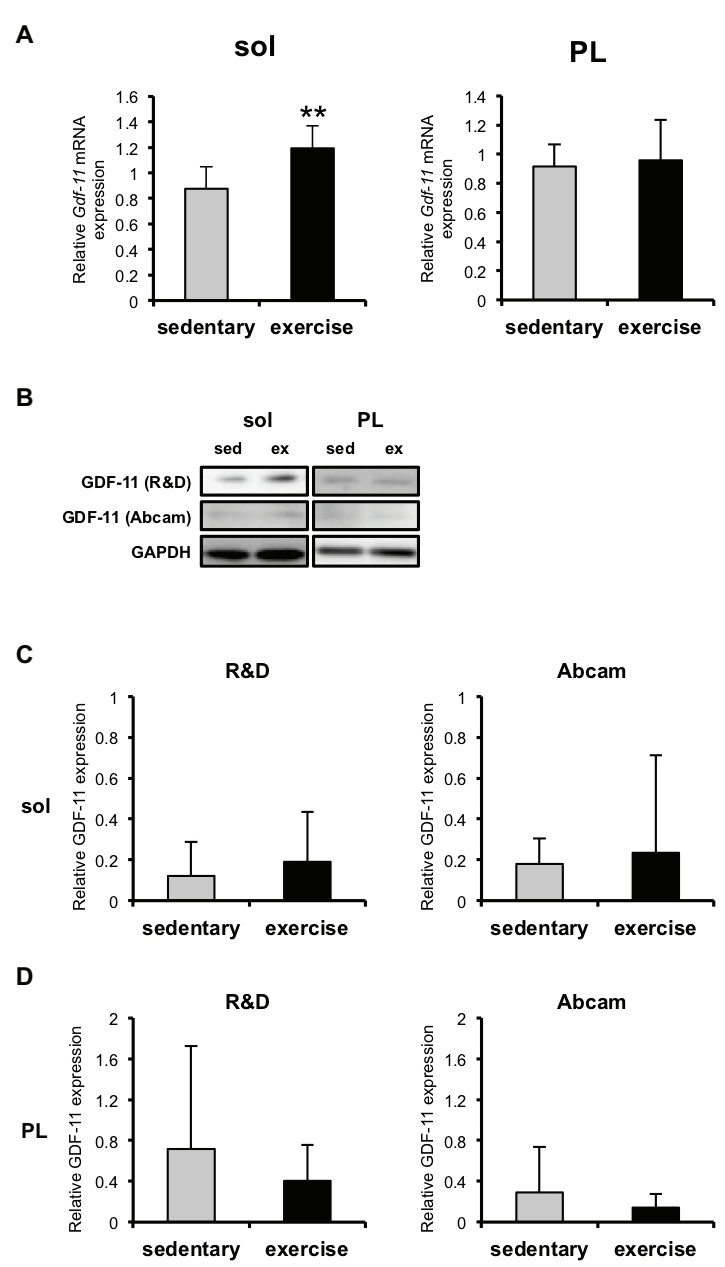
GDF11 mRNA and protein expression levels in skeletal muscles of aged sedentary and exercised mice. **(A)** *Gdf11* mRNA expression in soleus and plantaris of aged sedentary (*n* = 5) and aged exercised mice (*n* = 6). **(B)** GDF11 protein detected with western blotting with two different antibodies purchased from R&D Systems and Abcam. **(C)** Quantitative data for GDF11 protein levels in soleus and plantaris of aged sedentary (*n* = 4) and aged exercised mice (*n* = 6) detected with the R&D anti-GDF11 antibody. **(D)** Quantitative data for GDF11 protein levels in soleus and plantaris of aged sedentary (*n* = 5) and aged exercised mice (*n* = 6) detected with the Abcam anti-GDF11 antibody. Data are presented as means ± SD. ^**^p < 0.01 vs. the sedentary group.

*Gdf11* mRNA was upregulated in the soleus of the aged mice after 6 weeks of treadmill running (Figure 5A). In contrast, *Gdf11* mRNA was not affected by exercise training in the plantaris (Figure 5A). The GDF11 protein levels did not change in either the soleus or plantaris in response to exercise training (Figures 5B–D).

## Discussion

The biological role of GDF11 in skeletal muscle has not been clarified. In this study, we examined whether GDF11 is associated with exercise training in aged skeletal muscle. We first determined whether the age-related loss of muscle mass was associated with GDF11 expression in skeletal muscle. The skeletal muscle mass in the aged (22- to 26-month-old) mice was significantly lower than that in the young (5-month-old) mice. However, we observed no changes in GDF11 expression in the skeletal muscle with aging. The expression of GDF11 in skeletal muscle was previously investigated in the gastrocnemius and quadriceps muscles (Egerman et al., 2015; Poggioli et al., 2016; De Domenico et al., 2017), which are composed of both slow- and fast-twitch muscle fibers. Previous studies have reported that *Gdf11* mRNA in those muscles increased (Egerman et al., 2015; De Domenico et al., 2017) or did not change (Poggioli et al., 2016) with aging. We measured *Gdf11* mRNA expression in two distinct skeletal muscles; the soleus (dominantly composed of slow-twitch fibers) and the plantaris (dominantly composed of fast-twitch fibers) and observed that the *Gdf11* mRNA levels in both muscles were identical (data not shown). Consistent with a previous study (Poggioli et al., 2016), we observed that *Gdf11* mRNA levels in the soleus and plantaris did not change with aging. These findings suggest that *Gdf11* mRNA expression may not be associated with either aging or muscle-fiber type.

It has been demonstrated that the anti-GDF11 antibody (Abcam) used in previous studies detects not only GDF11 but also myostatin (Egerman et al., 2015; Smith et al., 2015). Using this antibody, previous studies have reported that circulating GDF11 dimers were either increased (Egerman et al., 2015) or unchanged in aged mice (Poggioli et al., 2016), whereas GDF11 monomers were reduced by aging (Egerman et al., 2015; Poggioli et al., 2016). In contrast, recent studies have reported that another anti-GDF11 antibody (R&D Systems) may be able to detect GDF11 without also detecting myostatin (Smith et al., 2015; De Domenico et al., 2017). Using this antibody, Domenico et al. reported that GDF11 dimers increased in the quadriceps muscle with aging (De Domenico et al., 2017). In the present study, we used antibodies purchased from both Abcam and R&D Systems. We observed that the levels of GDF11 dimers did not change with aging in either the soleus or plantaris muscles. This discrepancy with former studies could be attributable to the different muscle samples examined. Our findings suggest that not only *Gdf11* mRNA but also GDF11 protein levels may not be affected by aging in either slow or fast muscles.

A number of studies have reported that exercise training, including treadmill and voluntary wheel running, ameliorated age-related skeletal muscle loss in mice (Sakakima et al., 2004; Soffe et al., 2016). Given that the administration of recombinant GDF11 improved muscle function, including muscle strength and capacity for endurance exercise, in aged mice (Sinha et al., 2014), it could be speculated that GDF11 mediates the positive effects of exercise on sarcopenia. In this study, we investigated whether the effects of exercise training on sarcopenia involve changes in GDF11 expression in skeletal muscles. We observed that 6 weeks of treadmill running upregulated *Gdf11* mRNA in the soleus, but not in the plantaris, in aged mice. Considering that the transcriptional profile in skeletal muscle differs according to the muscle-fiber type (Terry et al., 2018) and changes differently in response to exercise (Raue et al., 2012; Murach et al., 2014), the transcriptional regulation of *Gdf11* in response to treadmill running might also differ between slow- and fast-twitch muscles. A recent study reported that 12 weeks of progressive rotarod training increased GDF11 protein expression in the quadriceps muscles in young mice (3 months old) but not in aged mice (18 months old) (De Domenico et al., 2017). Our data are the first to demonstrate a difference in GDF11 expression between slow- and fast-twitch muscles in response to exercise, although the biological significance must be investigated in future studies.

The treadmill exercise training conducted in this study did not increase the muscle mass of the aged mice, whereas it increased the CSAs of the muscle fibers. We speculate that exercise training might not only increase the muscle-fiber CSA but also reduce non-muscle tissues in the skeletal muscle, such as intramuscular adipose tissue, which usually increases with aging (Marcus et al., 2012). These might explain the observed discrepancy between the muscle mass and the fiber CSA. It has also been reported that aged mice refuse to run on a treadmill at over 20 m/min (Schefer and Talan, 1996), and we observed that the aged mice could not continue running at speeds exceeding 16 m/min in this study. Although the speed of the treadmill exercise was difficult for the aged mice, the exercise training frequency (once a day) might have been insufficient to induce changes in their muscle mass during the 6 weeks of the experimental period. Increased frequency of treadmill running exercise might be required because a previous study reported that the gastrocnemius muscle mass in 50-week-old senescence-accelerated mice increased after the treadmill running was performed twice a day (Sakakima et al., 2004).

## Conclusion

In summary, we have shown that GDF11 expression did not differ with aging or muscle-fiber type, but we did demonstrate that treadmill exercise training increased *Gdf11* mRNA in a predominantly slow-twitch muscle, specifically in aged mice.

## Data Availability

All datasets generated for this study are included in the manuscript and/or the Supplementary Files.

## Ethics Statement

All the mice were cage-housed in a temperature-controlled (21°C) environment under a 12-h light/12-h dark cycle, with free access to food and water according to the Guideline for Experimental Animal Care issued by the Prime Minister’s Office of Japan. The animal protocols were approved by the Animal Care and Use Committee of the University of Tokyo and Waseda University.

## Author Contributions

ML and TA designed the study. ML and SO performed the experiments. All authors contributed to the critical data analysis. KS, TU, and TA supervised the study. ML and TA wrote the manuscript, and all authors approved the final manuscript for publication.

### Conflict of Interest Statement

The authors declare that the research was conducted in the absence of any commercial or financial relationships that could be construed as a potential conflict of interest.
