# Supplementary material for: Effects of Exercise Training on Growth and Differentiation Factor 11 Expression in Aged Mice
Source: Front Physiol. 2019 Jul 31;10:970. doi: 10.3389/fphys.2019.00970 (PMC6684741; doi:10.3389/fphys.2019.00970)
Supplement: Supplementary file 1 [file Table_1.DOCX]

Supplementary table. Treadmill running protocol

| Week | | Inclination | Speed | Time | Total time |
| --- | --- | --- | --- | --- | --- |
| 1st | Day 1 | 5% | 10 m/min | 10 min | 10 min |
|  | Day 2 | 5% | 10 m/min | 5 min | 15 min |
|  |  |  | 11 m/min | 10 min |  |
|  | Day 3 | 10% | 10 m/min | 5 min | 15 min |
|  |  |  | 11 m/min | 10 min |  |
|  | Day 4 | 10% | 10 m/min | 2 min | 16 min |
|  |  |  | 11 m/min | 14 min |  |
|  | Day 5 | 10% | 11 m/min | 14 min | 16 min |
|  |  |  | 12 m/min | 2 min |  |
| 2nd | Day 1 | 10% | 11 m/min | 5 min | 17 min |
|  |  |  | 12 m/min | 12 min |  |
|  | Day 2 | 10% | 12 m/min | 17 min | 17 min |
|  | Day 3 | 10% | 12 m/min | 12 min | 17 min |
|  |  |  | 13 m/min | 5 min |  |
|  | Day 4 | 10% | 12 m/min | 5 min | 17 min |
|  |  |  | 13 m/min | 12 min |  |
|  | Day 5 | 10% | 13 m/min | 17 min | 17 min |
| 3rd | Day 1 | 10% | 13 m/min | 15 min | 18 min |
|  |  |  | 14 m/min | 3 min |  |
|  | Day 2 | 10% | 13 m/min | 13 min | 18 min |
|  |  |  | 14 m/min | 5 min |  |
|  | Day 3 | 10% | 13 m/min | 8 min | 18 min |
|  |  |  | 14 m/min | 10 min |  |
|  | Day 4 | 10% | 13 m/min | 3 min | 18 min |
|  |  |  | 14 m/min | 15 min |  |
|  | Day 5 | 10% | 14 m/min | 18 min | 18 min |
| Week | | Inclination | Speed | Time | Total time |
| 4th | Day 1 | 10% | 14 m/min | 16 min | 19 min |
|  |  |  | 15 m/min | 3 min |  |
|  | Day 2 | 10% | 14 m/min | 14 min | 19 min |
|  |  |  | 15 m/min | 5 min |  |
|  | Day 3 | 10% | 14 m/min | 9 min | 19 min |
|  |  |  | 15 m/min | 10 min |  |
|  | Day 4 | 10% | 14 m/min | 4 min | 19 min |
|  |  |  | 15 m/min | 15 min |  |
|  | Day 5 | 10% | 15 m/min | 19 min | 19 min |
| 5th | Day 1 | 10% | 15 m/min | 17 min | 20 min |
|  |  |  | 16 m/min | 3 min |  |
|  | Day 2 | 10% | 15 m/min | 15 min | 20 min |
|  |  |  | 16 m/min | 5 min |  |
|  | Day 3 | 10% | 15 m/min | 10 min | 20 min |
|  |  |  | 16 m/min | 10 min |  |
|  | Day 4 | 10% | 15 m/min | 7 min | 20 min |
|  |  |  | 16 m/min | 13 min |  |
|  | Day 5 | 10% | 15 m/min | 5 min | 20 min |
|  |  |  | 16 m/min | 15 min |  |
| 6th | Day 1 | 10% | 15 m/min | 3 min | 20 min |
|  |  |  | 16 m/min | 17 min |  |
|  | Day 2 | 10% | 15 m/min | 2 min | 20 min |
|  |  |  | 16 m/min | 18 min |  |
|  | Day 3 | 10% | 15 m/min | 1 min | 20 min |
|  |  |  | 16 m/min | 19 min |  |
|  | Day 4 | 10% | 16 m/min | 20 min | 20 min |
|  | Day 5 | 10% | 16 m/min | 20 min | 20 min |
